# Impact of Acupuncture on Human Metabolomic Profiles: A Systematic Review

**DOI:** 10.3390/metabo14100542

**Published:** 2024-10-11

**Authors:** Hongjin Li, Hannah Choi, Madelyn C. Houser, Changwei Li, Tingting Liu, Shuang Gao, Katy Sullivan, Judith M. Schlaeger

**Affiliations:** 1 College of Nursing, University of Illinois Chicago, Chicago, IL 60612, USA; hchoi213@uic.edu (H.C.); ksulli33@uic.edu (K.S.); jschlaeg@uic.edu (J.M.S.); 2 University of Illinois Cancer Center, Chicago, IL 60612, USA; 3 Nell Hodgson Woodruff School of Nursing, Emory University, Atlanta, GA 30322, USA; madelyn.elizabeth.crawford@emory.edu; 4 School of Public Health, Tulane University, New Orleans, LA 70112, USA; cli8@tulane.edu; 5 College of Nursing, Florida State University, Tallahassee, FL 32306, USA; tliu5@fsu.edu; 6 College of Medicine, University of Illinois Chicago, Chicago, IL 60612, USA; sgao20@uic.edu

**Keywords:** acupuncture, complementary and integrative health, human, mechanisms, metabolomics, pathway, traditional Chinese medicine, systematic review

## Abstract

Background/Objectives: Metabolomics provides insights into the biological underpinnings of disease development and treatment. This systematic review investigated the impact of acupuncture on metabolite levels and associated metabolic pathways using a metabolomic approach. Methods: Five databases (i.e., PubMed, Embase, Scopus, CINAHL, and Cochrane Central) were searched using terms such as “acupuncture” and “metabolites” to retrieve relevant journal articles published through January 2024. Studies utilizing mass spectrometry or nuclear magnetic resonance were included. Risk of bias was evaluated using the Cochrane Risk of Bias tool and the Newcastle–Ottawa scale. Metabolic pathway analysis was conducted using MetaboAnalyst 6.0 to identify common significant pathways affected by acupuncture. Additionally, subgroup pathway enrichment analysis identified metabolites significantly altered in more than two studies. Results: Among 4019 articles, 22 studies met inclusion criteria, examining changes in metabolomic biomarkers before and after acupuncture for various diseases and symptoms. A total of 226 metabolites showed significant changes, with 14 common metabolites altered in more than two studies (glutamine, androsterone glucuronide, choline, citric acid, decanoylcarnitine, estrone, glutathione, glycine, hypoxanthine, lactic acid, pyruvic acid, serine, proline, and sn-glycero-3-phosphocholine). Common pathways affected by acupuncture were glycine, serine, and threonine metabolism, glutathione metabolism, arginine biosynthesis, and glyoxylate and dicarboxylate metabolism. Conclusions: This review provides insights of the metabolomic mechanisms underlying acupuncture, highlighting its impact on specific metabolic pathways. Recognizing these changes can enhance acupuncture’s effectiveness and support the development of personalized treatments. The findings underscore metabolomics as a valuable tool for understanding and optimizing acupuncture for various diseases and symptoms.

## 1. Introduction

Acupuncture, a widely utilized complementary and integrative health (CIH) therapy, is deeply rooted in the principles of Traditional Chinese Medicine (TCM) [1]. Acupuncture has garnered considerable popularity, with an estimated over 10 million acupuncture treatments administered annually in the United States [2]. The overarching theory of TCM centers on the concept of yin and yang, which are mutually dependent and complementary forces. Health is maintained when these forces are in balance, but when they become imbalanced, disease occurs [3]. Qi is the vital energy that circulates throughout the body’s pathways, called meridians [4]. A balanced and unimpeded flow of qi is necessary for physical, emotional, and mental health [5,6]. Needling at specific body points is thought to restore the balance of yin and yang, and thus the body’s homeostasis [5,6]. A growing number of evidence has demonstrated the efficacy of acupuncture in treating not only chronic pain [7], but also various diseases and symptoms, such as side effects of cancer treatments, ischemic chest pain [8], and neuropsychiatric symptoms in Parkinson’s disease [9].

Emerging research is beginning to reveal the mechanisms of acupuncture and biological correlates of acupoints. Findings show specific influences of acupuncture on the central nervous system, including the modulation of neurotransmitters and the activation of specific brain regions implicated in pain regulation and homeostasis [10]. The effect of acupuncture on the immune system has been shown to regulate innate and adaptive inflammatory responses [11]. Many studies investigating the mechanisms of acupuncture have focused on one organ or organ system. However, there is a growing recognition of the need to consider acupuncture’s multiple and holistic effects on the body. Because acupuncture, a modality of TCM, is rooted in the concept of balance and harmony within the body, it is therefore essential to comprehend the mechanisms of acupuncture through a multi-organ, multi-system, systemic approach [12]. This approach is consistent with the National Institutes of Health (NIH), National Center for Complementary and Integrative Health (NCCIH) 2021–2025 Strategic Plan and its focus on personalized medicine and whole person health [13].

Analytical methods in systems biology can be used to examine complex processes involved in the human body’s reactions to medical interventions [14,15]. Metabolomics, a powerful analytical approach aimed at profiling small molecule metabolites within biological systems, offers a unique avenue for comprehending the mechanisms of acupuncture [16]. This methodology can provide insights of a comprehensive picture of biochemical changes induced by acupuncture, giving a snapshot of the physiological alterations occurring in response to treatment. Metabolomics allows for the exploration of intricate metabolic pathways influenced by acupuncture, elucidating its impact on cellular metabolism, energy regulation, and biochemical signaling cascades [17]. For example, studies have provided compelling evidence of acupuncture’s influence on tyrosine and tryptophan biosynthesis and glutathione metabolism in the treatment of fatigue and depression [18,19]. Through metabolomic analyses, researchers can unravel the complex interplay between acupuncture stimulation and physiological responses at the molecular level, ultimately enhancing our understanding of its therapeutic mechanisms to inform personalized treatment strategies.

While there have been reviews that have investigated the alterations in metabolite profiles with acupuncture, most have focused on animal models [20]. To the best of our knowledge, there has been no comprehensive systematic review of studies assessing metabolic profile changes pre- and post-acupuncture in humans. Therefore, we conducted this systematic review of human studies to investigate the impact of acupuncture on levels of metabolites and the metabolic pathways with which they are associated. We also compared the study design and methodologies used in the included metabolomics studies.

## 2. Methods

### 2.1. Design

This systematic review was conducted following the Preferred Reporting Items for Systematic Reviews and Meta-Analyses 2020 (PRISMA 2020) Guidelines for Reporting Systematic Reviews [21]. We consulted the Cochrane Handbook for Systematic Reviews of Interventions for guidance on standard systematic review methods [22]. This review protocol was registered in PROSPERO (ID: CRD42024503656).

### 2.2. Search and Retrieval Strategies

A total of five databases, including PubMed, Embase, Scopus, CINAHL, and Cochrane Central Register of Controlled Trials, were searched to identify relevant journal articles published through January 2024. Manual searches of the reference lists of publications were also conducted to identify additional relevant articles. The research team used these key search terms: acupuncture (“acupuncture*” or “electroacupuncture” or “acupuncture therapy” or “acupuncture analgesia” or “acupuncture, ear” or “pharmacoacupunctur*” or “acupotom*” or “auriculoacupunctur*” or “auriculotherap*” or “needle moxibustion” or “acupoint*” or “needling” or “trigger point*”) AND metabolites (“metabolite*” or “metabolomic*” or “metabolite marker*” or “metabolic profile*” or “metabolism*” or “biomarker*” or “biological marker*” or “endophenotype*” or “metabolome”) (Supplementary Materials: Table S1). All search terms were reviewed and confirmed by a health science librarian with expertise in search strategies.

### 2.3. Inclusion and Exclusion Criteria

The following criteria were used to select studies for inclusion: each study had to: (a) use samples (e.g., serum, plasma, saliva, urine) from human subjects; (b) use acupuncture (e.g., genuine body/ear acupuncture, electroacupuncture) as the primary intervention; (c) investigate changes of metabolite concentrations before and after the acupuncture intervention; (d) report metabolomics analysis techniques based on either gas or liquid chromatography-mass spectrometry (GC-MS/LC-MS) or nuclear magnetic resonance (NMR) spectroscopy applied to analyze metabolic changes; and (e) be published in a peer-reviewed, English-language journal. Studies were excluded from this review if they were: (a) protocol studies, reviews, letters to the editor, or conference abstracts; (b) animal studies; (c) studies that used methods other than mass spectrometry or nuclear magnetic resonance metabolomics methods to measure metabolites (e.g., studies that used biochemical analyzer kits were excluded). We did not place any restriction on the types of studies to be included, and thus randomized controlled trial (RCT), quasi-experimental, retrospective, and observational studies were all considered.

### 2.4. Selection of Studies

**Figure 1:**
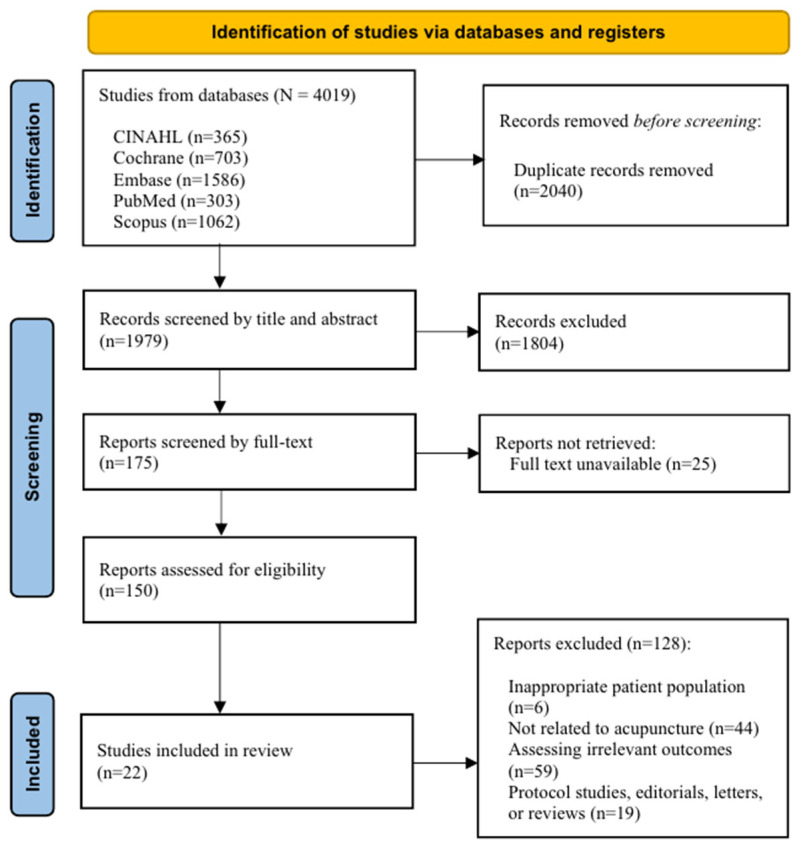
PRISMA flow diagram.

Two reviewers (H.C. and S.G.) independently screened potentially eligible articles with Covidence [23]. Specifically, articles were first screened by examining their titles and abstracts according to the eligibility criteria. Next, the full texts of potentially eligible articles were reviewed. Any inconsistencies between the two reviewers’ screening decisions were discussed with a third reviewer (H.L.) who made the final eligibility decision. The PRISMA flow diagram shown in Figure 1 describes the process of article selection.

### 2.5. Data Extraction and Analysis

After article selection, two reviewers (H.C. and S.G.) independently conducted data extraction by identifying study findings regarding metabolomics related to acupuncture and summarizing them in a matrix table. The following data were independently obtained and recorded in Microsoft Excel by the reviewers: (1) study characteristics (authors, year, country, participants, experimental and control groups, design, analytical platform and approach, sample and assessment time points, and acupuncture protocol) and (2) pathways (types of pathways, changes of metabolites, and statistical analysis method). A significance threshold of 0.05 was applied to determine statistically meaningful results during data extraction. Any inconsistencies in data extraction between the two reviewers were discussed with a third reviewer (H.L.) who made the final decision. A descriptive approach was used for the analysis of study data.

### 2.6. Pathway Analysis

To better understand the potential biological implications of the identified significant metabolites across studies, metabolic pathway analysis was performed in MetaboAnalyst 6.0. We imported a list of the significant metabolites with compound names. Metabolic pathway enrichment was performed using the following parameters: (1) enrichment analysis was performed using the hypergeometric test, (2) centrality was measured using relative betweenness, and (3) 80 human metabolic pathways in the Kyoto Encyclopedia of Genes and Genomes (KEGG) database were used as reference metabolic pathways. In addition, we identified common metabolites that exhibited significant changes following acupuncture in more than two of the studies. We performed a subgroup pathway enrichment analysis among these common metabolites. All *p* values were adjusted for multiple testing using the Benjamini–Hochberg false discovery rate (FDR) approach [24]. Pathways were considered enriched if the FDR < 0.05, three or more hits, and pathway impact score > 0.2.

### 2.7. Quality Assessment

The quality of the studies was assessed by two researchers (H.C., S.G.) using Cochrane Risk of Bias (RoB) 2 tool for RCTs [25] and the Newcastle–Ottawa Quality Assessment Scale (NOS) for non-randomized studies [26]. The Cochrane RoB 2 tool measures five domains, whether a study has: bias owing to the randomization process, deviations from the intended intervention, missing outcome data, errors in outcome measurement, or reporting only selected parts of the results. This tool includes three to seven questions for each domain and five level-of-response options for each question: “Yes”, “Probably yes”, “Probably no”, “No”, and “No information”. The tool includes hierarchical algorithms that convert responses to questions into domain-level judgments (i.e., “Low risk of bias”, “Some concerns”, and “High risk of bias”) and those into overall risk of bias judgements. The NOS measures three domains with a total of eight items: (1) selection of participants and ascertainment that outcome was followed by exposure, (2) comparability and control of the cohorts on the basis of the design, and (3) assessment of outcome. Each study is scored up to a total of nine points—a maximum of one point is given for each item within the Selection and Outcome domains, and maximum two points for Comparability. Seven or more points are considered “good”, two to six points are considered “fair”, and below one point is considered “poor” quality. If the two reviewers encountered any ambiguity in their assessments, the matter was discussed with a third reviewer (H.L.) to reach an appraisal decision.

## 3. Results

### 3.1. Study Identification

A total of 4019 articles were retrieved from the electronic databases. After removing 2040 duplicates, the remaining 1979 articles were screened by title and abstract based on the inclusion and exclusion criteria. Of these, 1804 were excluded for not meeting the criteria for the following reasons: having an inappropriate patient population, conducting an intervention that was not related to acupuncture or whose results did not allow identification of effects solely attributable to acupuncture, assessing outcomes other than metabolite changes and metabolomic pathways related to acupuncture effects, and taking the form of protocol studies, abstracts, or reviews. After reviewing the remaining full-text articles for eligibility, 22 studies were included in the final review (Figure 1).

### 3.2. Study Characteristics

In the studies reviewed, acupuncture was investigated in terms of its ability to treat a variety of diseases and symptoms: examples include migraine (*n* = 4) [27,28,29,30], psychoneurological symptoms including depression (*n* = 2) [19,31], hypertension (*n* = 2) [32,33], polycystic ovary syndrome (*n* = 2) [34,35], posterior circulation ischemia (*n* = 1) [36], obesity (*n* = 1) [37], fatigue (*n* = 1) [38], dry eyes (*n* = 1) [39], functional dyspepsia (*n* = 1) [40], functional constipation (*n* = 1) [41], kidney qi deficiency (*n* = 1) [42], sleep disorder (*n* = 1) [43], and stress urinary incontinence (*n* = 1) [44] (Table 1). Three studies recruited healthy people to explore the specific effect of acupuncture using the Zusanli acupoint [16,45,46].

The sample size for the studies ranged from 8 to 128. Acupuncture was used in 16 studies, while 5 studies used electroacupuncture, and 1 study used acupuncture combined with electroacupuncture. As for research designs employed, 11 studies were RCTs, 7 were quasi-experimental studies, and 4 were prospective cohort studies. For the 11 RCTs, 5 were two-arm trials comparing acupuncture with sham acupuncture (*n* = 3) [33,37,42] and with usual care (*n* = 2) [19,38]. Five studies were three-arm RCTs comparing acupuncture with sham acupuncture and healthy control (*n* = 3) [27,36], and with alternative therapies and usual care (*n* = 3) [34,35,41]. The remaining one RCT was a four-arm trial comparing acupuncture with sham, usual care, and healthy controls [29]. Each acupuncture study used a standardized acupuncture protocol, with acupuncture sessions ranging in number from one to 20.

### 3.3. Risk of Bias Assessment

To assess risk of bias, the Cochrane RoB 2 tool was used for the 11 RCTs, and the NOS was used for the remaining 11 non-randomized studies. The assessment results are shown in Table 2. Seven studies showed a low risk of bias, or good quality [28,31,32,35,37,39,43]. Thirteen studies presented some concerns regarding risk of bias, or fair quality [16,27,29,30,34,36,38,40,41,42,44,45,46]; these concerns generally involved potential bias caused by a less rigorous random assignment process, non-adherence to the intervention, insufficient control of confounding factors, and an inadequate quantity of follow-up data. Two studies showed a high risk of bias [19,33], which arose mainly from potential bias caused by non-adherence to the intervention and missing outcome data.

### 3.4. Metabolomic Approaches, Sample Type and Timing

For metabolomic approaches, most of the studies applied an untargeted approach (*n* = 13) [16,19,30,31,36,38,39,40,41,43,44,45,46], while nine studies used a targeted approach [27,28,29,32,33,34,35,37,42]. Mass spectrometry was predominantly used for evaluating novel metabolite biomarkers (*n* = 19) [16,19,28,29,30,31,32,33,34,35,36,37,39,41,42,43,44,45,46], followed by one proton-nuclear magnetic resonance analysis (1H-NMR) approach (*n* = 3) [27,38,40]. Most of the studies used human blood samples, including plasma (*n* = 6) [27,29,30,32,33,40] and serum (*n* = 7) [31,35,37,41,43,44,46]. Other studies used saliva (*n* = 2) [16,34], urine (*n* = 4) [19,36,38,45], tears (*n* = 1) [39], follicular fluid (*n* = 1) [42], and proton magnetic resonance spectroscopy imaging of the brain (*n* = 1) [28]. A fasting condition for sample collection was indicated in 11 studies [27,30,31,32,33,34,35,37,40,41,46], and a specific time for collecting samples after the acupuncture intervention was reported in eight studies [27,30,31,32,33,35,40,46]. With respect to the timing of sampling after the acupuncture intervention, we categorized studies into three categories: early (0–24 h after acupuncture, *n* = 3) [30,38,46], intermediate (0–3 days after acupuncture, *n* = 4) [36,37,40,42], and late (0–7 days after acupuncture, *n* = 1) [35].

### 3.5. Metabolites with Significant Changes after Acupuncture

All the studies reported a significant (*p* < 0.05) increase or decrease in metabolites after the acupuncture intervention (Table 3). In total, 226 unique metabolites were found to change significantly after acupuncture across the 22 studies (Supplementary Materials: Table S2). We identified 15 common metabolites that changed significantly after acupuncture in more than two of the studies across multiple studies with various sample types and study populations, including glutamine [27,45], androsterone glucuronide [35,45], choline [29,38], citric acid [27,38,44,45], decanoylcarnitine [37,42], estrone [35,36], glutathione [19,31], glycine [32,38,42], hypoxanthine [33,38], lactic acid [38,40], pyruvic acid [27,42], serine [37,39], proline [19,29], and sn-glycero-3-phosphocholine [42,46].

### 3.6. Metabolomic Pathways Related to Acupuncture

**Figure 2:**
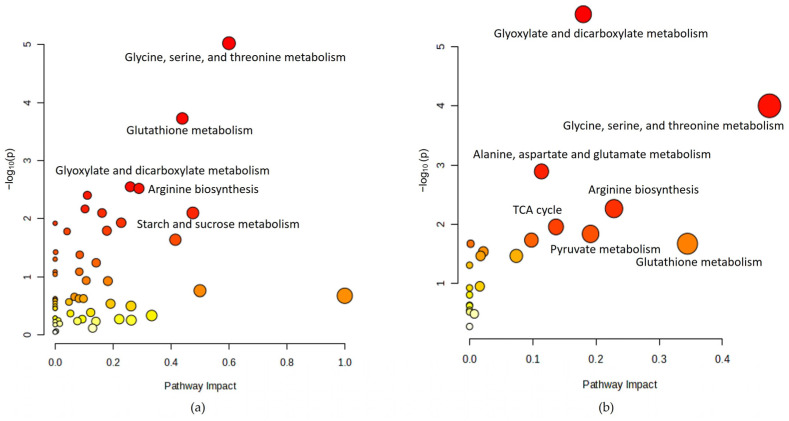
Metabolomic pathways related to acupuncture. (**a**) Pathway analysis with 226 significant metabolites; (**b**) pathway analysis with 14 common metabolites.

Across all significant metabolites identified from all the studies, all the enriched pathways are displayed in Figure 2a. Significant metabolomic pathways related to acupuncture treatment were glycine, serine, threonine metabolism (raw *p* < 0.001, FDR < 0.001, impact score = 0.60), and glutathione metabolism (raw *p* = 0.001, FDR = 0.007, impact score = 0.44). Other possible metabolomics pathways were arginine biosynthesis (raw *p* = 0.003, FDR = 0.06, impact score = 0.29), and glyoxylate and dicarboxylate metabolism (raw *p* = 0.003, FDR = 0.06, impact score = 0.26).

Across 15 common significant metabolites identified in more than two studies, all the enriched pathways are displayed in Figure 2b. Significant metabolomic pathways related to acupuncture treatment were glycine, serine, and threonine metabolism (raw *p* < 0.001, FDR = 0.004, impact score = 0.47). Results from all pathway analyses are listed in Supplementary Materials: Tables S3 and S4.

## 4. Discussion

Progress in mass spectrometry technology since 2010 has resulted in a growing body of metabolomics-focused research examining the metabolic responses to acupuncture. Nevertheless, the utilization of metabolomics to investigate the mechanism of acupuncture in human subjects is still in its early stages. In this systematic review, we identified 22 studies examining alterations in metabolomic biomarkers before and after acupuncture treatment for various diseases and symptoms. Due to the limited number of metabolomics studies on acupuncture, it is challenging to conduct a comprehensive review focusing on a homogeneous population. Nonetheless, this review elucidates that common metabolomic pathways are emerging in the treatment of various diseases and symptoms with acupuncture, and these pathways are interconnected and interact with each other. Results of the review suggest that acupuncture treatment mainly influences carbohydrate and amino acid metabolism. This study also underscores the significance of metabolomics as a potential tool for comprehending the mechanisms by which acupuncture may treat various diseases and symptoms.

### 4.1. Glycine, Serine, and Threonine Metabolism

Glycine is a simple amino acid involved in the synthesis of proteins, neurotransmitters, key molecules involved in antioxidant protection by glutathione, and one-carbon metabolism [47]. Glycine is interconnected with serine and threonine. Serine is generated via glycine and is then converted into pyruvate by the enzyme serine dehydratase. Threonine is associated with energy metabolism and promotes the cellular defense function of the immune system [48]. Dysregulation of the glycine, serine, and threonine metabolic pathway has been linked to various disorders, including metabolic diseases, neurological disorders, and cancer [42,49].

In the articles reviewed, glycine levels were reduced in follicular fluids (due to increased glycine metabolism into guanidinoacetic acid) after acupuncture for women who were diagnosed with infertility and underwent in vitro fertilization (IVF) assisted pregnancy [42]. Yang et al. [32] also observed reduced glycine levels post-acupuncture, with higher levels in hypertensive patients compared to healthy controls before treatment. A reduction in glycine levels may arise from a combination of decreased biosynthesis, increased catabolism, or heightened urine excretion simultaneously. Interestingly, Ma et al. [38] found that glycine levels were significantly increased in urine immediately after acupuncture for athletes that had fatigue induced by physical exercise. Serine levels were increased after acupuncture in serum samples of obese premenopausal women and were increased in tear samples of patients with dry eye diseases [37,39]. Pyruvic acid decreased after acupuncture in patients with migraine and women who underwent IVF-assisted pregnancy [27,42]. Our pathway analysis suggests that glycine, serine, and threonine metabolism was the common metabolic pathway disturbed by acupuncture across different disease conditions. Further studies are required to validate whether acupuncture leads to upregulation or downregulation of glycine, serine, and threonine metabolism across different disease conditions, different tissues or biofluids, and different times post-acupuncture treatment.

### 4.2. Glyoxylate and Dicarboxylate Metabolism

Glyoxylate and dicarboxylate metabolism are interconnected metabolic pathways involved in the utilization of two-carbon compounds, such as glyoxylate and various dicarboxylates that are carbon sources for energy production and biosynthesis. The key enzyme within the glyoxylate pathway is isocitrate lyase, responsible for catalyzing the breakdown of isocitrate into glyoxylate and succinate [50]. This process circumvents the two oxidative stages of the tricarboxylic acid (TCA) cycle, enabling organisms to utilize acetyl-CoA obtained from fatty acids or other origins for carbohydrate synthesis [51]. Dicarboxylate metabolism involves the use of dicarboxylic acids like succinate, malate, and fumarate for carbon sources. These acids can enter the TCA cycle and serve as intermediates for energy production or biosynthesis. Dysfunctional energy metabolism and altered citric acid cycle activity may lead to increased levels of citric acid, found in patients with migraines, stress urinary incontinence, fatigue, and polycystic ovary syndrome (PCOS)-related obesity [27,34,38,44,45].

In the articles reviewed key metabolites related to glyoxylate and dicarboxylate metabolism including succinic acid, isocitric acid, and citric acid were significantly perturbed in five studies [27,34,38,44,45]. Citric acid, and succinic acid were significantly decreased after acupuncture in plasma samples of women with migraine [27], in serum samples of women with stress urinary incontinence [44], and in serum samples of women with PCOS-related obesity [34]. Also, acupuncture effectively restored and increased citric acid, pyruvate, and glycine levels to their normal state, which had been disrupted by exhaustive physical exercise in young male athletes [38]. These studies provide evidence that acupuncture could regulate carbohydrate metabolism. More research is needed to establish a direct causal relationship between acupuncture and an increase in carbohydrate metabolism and to understand the specific mechanisms involved.

### 4.3. Glutathione Metabolism

Glutathione is a tripeptide composed of three amino acids: glutamate, cysteine, and glycine. It acts as a powerful antioxidant, protecting cells from oxidative damage caused by reactive oxygen species. Studies have found associations between reduced glutathione levels and the presence of depression and fatigue [52,53]. By replenishing glutathione levels through supplementation or lifestyle interventions, individuals may experience improvements in fatigue and depression symptoms [54].

In the articles reviewed, increased glutathione was found in two studies after acupuncture. The glutathione level was increased in urine samples of patients with depression and increased in serum samples of breast cancer survivors experiencing psychoneurological symptoms after acupuncture [19,31]. Evidence from animal studies suggest that acupuncture can inhibit oxidative stress by increasing glutathione levels, thus enhancing the activity of antioxidative enzymes, such as glutathione peroxidase [55,56]. Similarly, increased glutathione peroxidase and glutathione were found in 40 obese and overweight individuals after acupuncture, suggesting that acupuncture could enhance the antioxidant defense system [57]. Future investigations could focus on conducting targeted pathway analyses and assessing changes in absolute concentrations of glutamate, glutathione, and related antioxidative enzymes before and after acupuncture to validate these findings.

### 4.4. Arginine Biosynthesis

Arginine biosynthesis is a vital metabolic pathway responsible for the production of arginine, a semi-essential amino acid. In humans, arginine can be synthesized via two main pathways: the urea cycle and the ornithine biosynthetic pathway [58]. The conversion of glutamate to ornithine is a crucial step in the urea cycle and ornithine biosynthetic pathway, both of which contribute to arginine production in the body. Glutamate is an amino acid that serves as a neurotransmitter in the central nervous system, is involved in energy metabolism, and serves as a precursor for the synthesis of other amino acids and molecules [59]. Glutamate is a critical neurotransmitter involved in the pathophysiology of migraine headaches and central sensitization due to its excitatory action on nociceptive neurons in the trigeminovascular system [60]. Lower levels of glutamatergic metabolites have been associated with depression and fatigue [61], contrasting with higher glutamate levels observed in migraine sufferers [60].

In this review, studies found that migraine patients often exhibit elevated serum levels of arginine during non-headache periods, along with disturbances in arginine, glutamate, citrulline, and aspartate [30]. Increased citrulline and decreased glutamate were found in patients with migraine after acupuncture [30]. Similarly, acupuncture can reduce glutamate levels and modulate glutamate receptors and excitatory amino acid transporter expression [62], thereby alleviating neuropsychiatric and migraine symptoms.

### 4.5. Acupuncture’s Therapeutic Effects: Link Metabolomics Changes to Clinical Outcomes

Most studies included in this review primarily focused on the changes in metabolites levels before and after acupuncture treatment, only five studies examined the correlation between metabolomic changes and clinical outcomes [27,28,29,32,44]. Several biomarkers were found to monitor the therapeutic effects of acupuncture on migraine. Gu et al. (2018) found that changes in the N-acetylaspartate/creatine ratio were linked to reduced headache intensity [28]. Liu et al. (2022) found that Biliverdin Reductase B and Flavin Adenine Dinucleotide were positively correlated with pain intensity, whereas alpha-D-glucose, citrulline, and L-noradrenaline showed negative correlations with pain intensity after acupuncture [30]. Similarly, Li et al. (2023) found that pain intensity in migraines was negatively correlated with 4-Oxoproline and positively correlated with metabolites such as corticosterone and eicosapentaenoic acid in migraine after acupuncture treatment [29]. These findings suggest that alterations in these metabolites may reflect reductions in headache severity, potentially serving as biomarkers for assessing the therapeutic effects of acupuncture on migraine.

In addition, octanoic acid (OA) and myo-inositol (MI) may serve as indicators of acupuncture’s antihypertensive effects. Yang et al. (2016) demonstrated that the reduction in systolic blood pressure was positively correlated with changes in OA and negatively correlated with MI changes in patients with hypertension after acupuncture treatment [32]. In Zhang et al.’s study (2020), several metabolites—such as utantriol, 3,4-dihydroxybutanoic acid, succinic acid, 1-deoxypentitol, citric acid, 3-hydroxybutyric acid, and hydracrylic acid—were positively correlated with clinical outcomes (measured by urine leakage in the 1-h pad test, 72-h incontinence episodes, and the ICIQ-SF score) [44]. On the other hand, psicose showed a negative correlation with these clinical indices. These findings suggest that specific metabolite changes may reflect the severity of urinary incontinence, potentially serving as biomarkers to monitor acupuncture’s therapeutic impact on the condition.

To understand acupuncture’s therapeutic effects in clinical outcomes, it is essential to examine how metabolite changes correlate with clinical improvements. If certain metabolites consistently decrease after acupuncture and are tied to positive outcomes, they could serve as reliable biomarkers. Several statistical approaches can be used to explore these associations, such as correlation analyses, mixed-effects models, mediation analysis, and machine learning techniques (i.e., random forests). Future studies can use these statistical approaches to deepen our understanding of how metabolite changes reflect the therapeutic effects of acupuncture.

### 4.6. Issues and Challenges in Acupuncture Metabolomics Research

Metabolomics studies are influenced by numerous factors including variations in subject state and in methods for sample collection and processing and for data collection, reflecting the complexity of biological systems and the intricacies of experimental design and analysis. This reduces the likelihood of identifying identical metabolites and metabolic pathways from one study to another. Consideration of these factors is crucial for designing robust metabolomics studies, interpreting results accurately, and drawing meaningful conclusions about biological processes and responses.

#### 4.6.1. Biological and External Factors

Inherent differences in metabolite profiles between individuals, such as age and sex, are significant factors that can influence metabolomics research. Metabolite profiles tend to change with age due to various physiological alterations, including changes in metabolism, hormonal levels, and organ function [63]. These age-related variations can impact baseline metabolite levels and responses to environmental stimuli or interventions. Sex plays a critical role in shaping the metabolic response to acupuncture. Differences in hormones, pain perception, immune response, and baseline metabolite profiles between men and women can lead to distinct outcomes in acupuncture metabolomics studies [64]. For example, Gu et al. [28] found that among participants with cervicogenic headaches, females had significantly lower NAA/Cr levels compared to males after acupuncture treatment (*p* = 0.024). It is important to control for these factors during study design and data analysis. External factors, such as disease status, diet, exercise, medications, and other lifestyle factors can also influence metabolic profiles. The wide range of disease conditions represented by the participants in the reviewed studies reduces the likelihood of identifying consistent metabolic effects of acupuncture across studies. While the identification of common metabolomic pathways holds promise for understanding the general therapeutic mechanisms of acupuncture, the heterogeneity among study populations underscores the need for robust study designs and larger, more standardized cohorts to achieve conclusive insights.

#### 4.6.2. Study-Related Factors

Consistency in sample collection and handling, analytical techniques, and experimental design are crucial in metabolomics research. Fasting status and standardized time intervals for biospecimen collection, such as early morning, are essential for collecting optimal data with inter-subject variation minimized. Fasting allows researchers to examine basal metabolism and metabolic homeostasis without external dietary influences [65], providing a reliable reference for evaluating metabolic responses to acupuncture. However, many studies we reviewed did not specify the exact time of sample collection after acupuncture. Immediate and prolonged sampling intervals may yield differing metabolite levels. Therefore, researchers should consistently incorporate and quantify these parameters in study designs to enhance comparability between studies. Rigorous clinical research methodologies like RCT designs should be employed in addition to biomedical analyses. Valid control arms should be used to rule out the possibility of a non-specific/placebo effect.

Untargeted metabolomic profiling poses a risk of false discovery. Treating statistically differential features as potential biomarkers without verifying their identities can be risky. To reduce irrelevant false positive biomarkers, employing a suitable fold-change threshold, conducting multicenter studies, and validating results with an independent patient cohort are strategies to consider [66]. It is essential to perform a targeted metabolomics analysis to quantify absolute values following the identification of potentially significant pathways or metabolites in untargeted metabolomic profiling.

### 4.7. Limitations and Strength

While this review has provided valuable insights into the state of knowledge of metabolomics changes after acupuncture, there are several limitations to acknowledge. We performed the metabolic pathway analysis by pooling significant metabolites identified from the reviewed studies. Due to the limited research on metabolomics and acupuncture, our ability to conduct pathway analyses with a homogeneous set of studies based on specific diseases, populations, or specimen types was constrained by insufficient evidence. We believe that the mechanisms underlying acupuncture’s efficacy in treating various diseases or symptoms have both similarities and differences. The similarities lie in the fact that acupuncture can promote the body’s self-regulation and healing by adjusting Qi, blood circulation, balancing yin and yang, and regulating organ functions. Regardless of the specific disease, acupuncture can adjust the body’s physiological state by promoting the balance of the neuroendocrine system, alleviating pain, improving blood circulation, which are significant for various diseases. This review aimed to give only an insight into the common metabolomic pathways underlying acupuncture. Caution should be exercised when interpreting results across studies, considering the potential differences in underlying mechanisms, and the heterogeneous nature of the population and conditions being studied. In addition, there is substantial variation in the acupuncture protocols used across the studies reviewed (e.g., electroacupuncture vs. manual acupuncture). This variability could significantly influence the metabolomic outcomes. Future reviews can conduct subgroup analysis to interpret how the acupuncture protocol (e.g., electroacupuncture vs. manual acupuncture) and disease type impact the metabolic pathway. Furthermore, many studies lacked rigorous reporting of metadata related to mass spectrometry and statistical measures, making it difficult to assess the confidence in metabolite annotations. For example, only a few studies provided data on retention time, peak areas, fold changes, *p*-values, and false discovery rates. Moreover, significant pathways were summarized using inconsistent criteria, hindering the comparison of findings. These metrics are crucial for identifying meaningful changes in metabolites and assessing their clinical relevance. Therefore, we suggest that future studies follow the Metabolomics Standards Initiative guidelines [67] to report study results. Researchers are recommended to report: (1) metadata relative to mass spectrometry (i.e., ionization source, acquisition mode, data acquisition parameters); (2) data pre-processing; (3) metabolite identification (four levels of metabolite identification), and (4) statistical methods for selecting metabolites associated with the variable of interest (i.e., adjusted *p*-value and FDR thresholds, any further curation of the selected feature pool).

## 5. Conclusions

This systematic review summarized the results of human acupuncture and metabolomics studies. The most common pathways impacted by acupuncture are glycine, serine, and threonine metabolism and glutathione metabolism. Recognizing significant changes in the metabolic pathways underlying acupuncture can help in leveraging acupuncture’s therapeutic potential and optimizing its delivery to enhance its effectiveness. Metabolomics offers insight into the biological underpinnings of disease development, treatment approaches, and the mechanisms of acupuncture. Future implications of metabolomics include an essential role in the advancement of personalized medicine and whole-person health, as well as acupuncture science [13].

## Figures and Tables

**Table 1:** Study characteristics (*N* = 22).

Author/ Year/ Country	Participants (Sample Size; Female % [Male/Female])	Experimental Group	Control Group	Research Design	Analytical Platform	Approach/ Sample Type	Fasting Condition	Sample Collection Timepoints *	Acupuncture Protocol
Gao 2023 China [27]	Women aged 20–45 with or without migraine (*N* = 50; 100% [0/50])	EA (*n* = 22, patients with migraine)	- Sham EA (*n* = 18, patients with migraine); - Healthy controls (*n* = 10).	RCT	1H-NMR	Targeted/ plasma	Yes (7:30–9:30 AM)	Pre- and post-acupuncture (not in detail)	20 sessions over 4 weeks, 5 times per week, 30 min/session
Gu 2018 China [28]	Patients aged 18–60 years, diagnosed with migraine or cervicogenic headache, and healthy controls (*N* = 44; 68.2% [14/30])	Acupuncture (*n* = 15, patients with migraine)	- Acupuncture (*n* = 15, patients with cervicogenic headache); - Sham acupuncture (*n* = 14, healthy controls).	Quasi-experimental	MRSI	Targeted/ MRI	NA	Pre- and post-acupuncture (not in detail)	5 sessions for a week, one session per day
Jedel 2011 Sweden [35]	Women aged 18–37 years with PCOS (*N* = 74; 100% [0/74])	Low-frequency EA (*n* = 29, patients with PCOS)	- Aerobic exercise (*n* = 30, patients with PCOS); - Usual care (*n* = 15, patients with PCOS)	RCT	GC/LC-MS	Targeted/ serum	Yes (7:30–8:30 AM)	Late: baseline, week 16, week 32 (within 1 wk)	14 sessions for 16 weeks, 1–2 times per week, 30 min/session
Ju 2016 China [36]	Patients diagnosed with or without PCI (*N* = 90; 64.4% [32/58])	Acupuncture with needle tip toward the contralateral paropia (*n* = 30, patients with PCI)	- Acupuncture with needle tip toward the prominentia laryngea (*n* = 30, patients with PCI); - Healthy controls (*n* = 30)	RCT	LC-MS	Untargeted/ urine	NA	Intermediate: pre- and post-acupuncture (at the second day after the final treatment)	14 sessions, 3–4 sessions per week
Kim 2021 South Korea [37]	Premenopausal overweight and obese adult women (*N* = 120; 100% [0/120])	Acupuncture with EA (*n* = 60, premenopausal overweight and obese women)	Sham MA with sham EA (*n* = 60, premenopausal overweight and obese women)	RCT	LC-MS	Targeted/ serum	Yes	Intermediate: pre- and post-acupuncture (within 3 days)	12 sessions for 6 weeks, twice per week, 30 min/session
Li 2020 China [19]	Patients aged 18–70 years with moderate depression (*N* = 60; 63.3% [22/38])	EA (*n* = 30, patients with depression)	Usual care (*n* = 30, patients with depression)	RCT	GC-MS	Untargeted/ urine	NA	Pre- and post-acupuncture (not in detail)	8 weeks, 3 times a week, 30 min/session
Li 2023 China [29]	Patients aged 18–55 years with or without migraine (*N* = 48; 60.4% [19/29])	Acupuncture (*n* = 12, patients with migraine)	- Sham acupuncture (*n* = 13, patients with migraine) - Usual care (*n* = 13, patients with migraine) - Healthy controls (*n* = 10)	RCT	LC-MS	Targeted/ plasma	NA	Pre- and post-acupuncture (not in detail)	6 sessions for 2 weeks, three times per week, 30 min/session
Li 2023 U.S. [31]	Breast Cancer Survivors with psychoneurological symptoms (*N* = 8; 100% [0/8])	Acupuncture (*n* = 8, patients with breast cancer)	NA	Cohort (prospective)	LC-MS	Untargeted/ serum	Yes (8:00–11:00 AM)	Pre- and post-acupuncture (not in detail)	10 sessions for 5 weeks, 30 min/session
Liu 2022 China [30]	Women with migraine without aura and healthy controls (*N* = 30; 100% [0/30])	Acupuncture (*n* = 20, patients with migraine)	Healthy controls (*n* = 10)	Quasi -experimental	LC-MS	Untargeted/ plasma	Yes (7:30–8:30 AM)	Early: pre- and post-24 h after acupuncture	12 sessions, 4 weeks, three times per week, 30 min/session
Ma 2015 China [38]	Young male athletes with fatigue induced by physical exercise (*N* = 14; 0% [14/0])	Acupuncture (*n* = 7, young male athletes)	Healthy controls (*n* = 7, young male athletes)	RCT	1H-NMR	Untargeted/ urine	NA	Early: before exercises, and 35 min after exercise (before acupuncture), and post-acupuncture	Single session, 30 min/session
Rao 2021 China [39]	Patients with dry eye disease (*N* = 18; 50.0% [9/9])	Acupuncture (*n* = 9, patients with dry eye disease)	Drug treatment (*n* = 9, patients with dry eye disease)	Quasi -experimental	LC-MS	Untargeted/ tear	NA	Pre- and post-acupuncture (not in detail)	10 sessions for 3 weeks, 30 min/session
Wu 2010 China [40]	Women with or without functional dyspepsia (*N* = 12; 100% [0/12])	Acupuncture (*n* = 6, women with functional dyspepsia)	Healthy controls (*n* = 6)	Quasi -experimental	1H-NMR	Untargeted/ plasma	Yes (8:00 AM)	Intermediate: pre- and post-acupuncture (second day)	6 sessions, 6 days
Wu 2023 China [41]	Patients with functional constipation (*N* = 19; 89.5% [2/17])	EA with low current intensity (*n* = 7, patients with functional constipation) EA with high current intensity (*n* = 6, patients with functional constipation)	Mosapride citrate tablet (*n* = 6, patients with functional constipation)	RCT	GC-MS	Untargeted/ serum	Yes	Pre- and post-acupuncture (not in detail)	16 sessions for 4 weeks, 3–5 times per week, 30 min/session
Xia 2023 China [42]	Women aged 35–42 years with kidney qi deficiency who underwent IVF-assisted pregnancy (*N* = 60; 100% [0/60])	Acupuncture (*n* = 30, women with kidney qi deficiency)	Sham acupuncture (*n* = 30, women with kidney qi deficiency)	RCT	MRM-MS	Targeted/ follicular fluids	NA	Intermediate: pre- and post-acupuncture (after 36 h)	3 times a week until the day of hCG injection
Yan 2013 China [45]	Healthy volunteers (*N* = 20; not reported)	Acupuncture (*n* = 20, healthy subjects)	NA	Cohort (prospective)	LC-MS	Untargeted/ urine	NA	Baseline, at day 7, 14 (not in detail)	14 sessions for 2 weeks, once a day.
Yang 2016 China [32]	Patients with essential hypertension (*N* = 128; 70.3% [38/90])	Acupuncture (*n* = 113, patients with essential hypertension)	Healthy controls (*n* = 15)	Quasi -experimental	MRM-MS	Targeted/ plasma	Yes (8:00–9:00 AM)	Pre- and post-acupuncture (not in detail)	6 weeks, 3 times a week, 30 min/session
Yang 2018 China [33]	Patients with essential hypertension, aged 45–75 (*N* = 13; 38.5% [8/5])	Acupuncture with active acupoint treatment (*n* = 5, patients with essential hypertension)	Sham acupuncture with inactive acupoint treatment (*n* = 8, patients with essential hypertension)	RCT	LC-MS	Targeted/ plasma	Yes (8:00–9:00 AM)	Pre- and post-acupuncture (not in detail)	6 weeks, 3 times a week, 30 min/session
Yang 2023 China [34]	Patients with PCOS related obesity (*N* = 60; 100% [0/60])	Acupuncture (*n* = 20, patients with PCOS related obesity)	- Western medicine (*n* = 20, patients with PCOS related obesity) - Acupuncture combined with Western medicine (*n* = 20, patients with PCOS related obesity)	RCT	LC-MS	Targeted/ serum	Yes	Pre- and post-acupuncture (not in detail)	6 weeks, 3 times a week
Yang 2023 China [43]	Patients with mobile phone addiction and sleep disorder and health controls (*N* = 12; not reported)	Acupuncture (*n* = 6, patients with mobile phone addiction and sleep disorder)	Healthy controls (*n* = 6)	Quasi -experimental	LC-MS	Untargeted/ saliva	NA	Pre- and post-acupuncture (not in detail)	7 sessions for a week (one day per session).
Zhang 2014 China [16]	Healthy male volunteers (*N* = 20; 0% [20/0])	Acupuncture (*n* = 20, healthy males)	NA	Cohort (prospective)	LC-MS	Untargeted/ saliva	NA	Pre- and post-acupuncture (not in detail)	14 sessions for 2 weeks, once a day, 30 min/session
Zhang 2016 China [46]	Healthy male volunteers (*N* = 20; 0% [20/0])	Acupuncture (*n* = 20, healthy males)	NA	Cohort (prospective)	LC-MS	Untargeted/ serum	Yes (5:00–7:00 AM)	Early: pre- and post-acupuncture (on the treatment completion day)	14 sessions for 2 weeks, once a day.
Zhang 2020 China [44]	Women with stress urinary incontinence (*N* = 50; 100% [0/50])	EA (*n* = 25, women with stress urinary incontinence)	Sham EA (*n* = 25, healthy controls)	Quasi -experimental	GC-MS	Untargeted/ serum	NA	Pre- and post-acupuncture (not in detail)	18 sessions for 6 weeks, 3 times per week

Note. 1H-NMR = 1H (proton)-nuclear magnetic resonance; EA = electroacupuncture; GC-MS = gas chromatography-mass spectrometry; IVF = in vitro fertilization; LC-MS = liquid chromatography-mass spectrometry; min = minute; MRI = magnetic resonance imaging; MRSI = magnetic resonance spectroscopy imaging; MRM-MS = Multiple Reaction Monitoring-Mass Spectrometry; NA = not assessed; PCI = posterior circulation ischemia; PCOS = polycystic ovary syndrome; RCT = randomized controlled trial. * Sample collection time points: early (0–24 h after acupuncture), intermediate (0–3 days after acupuncture), and late (0–7 days after acupuncture).

**Table 2:** Quality assessment: (**a**) Cochrane ROB2 (*n* = 11). (**b**) Newcastle–Ottawa Scale (*n* = 11).

**(** **a)**							
**Author/Year/** **Country**	**Study Design**	**Assignment to Intervention**	**Adhering to Intervention**	**Missing Outcome Data**	**Measurement of the Outcome**	**Selection of the Reported Result**	**Overall Risk of Bias**
Gao 2023 China [27]	RCT	Some concerns	Low	Low	Low	Low	Some concerns
Jedel 2011 Sweden [35]	RCT	Low	Low	Low	Low	Low	Low
Ju 2016 China [36]	RCT	Some concerns	Some concerns	Low	Low	Low	Some concerns
Kim 2021 South Korea [37]	RCT	Low	Low	Low	Low	Low	Low
Li 2020 China [19]	RCT	Low	Some concerns	High	Low	Low	High
Li 2023 China [29]	RCT	Some concerns	Some concerns	Low	Low	Low	Some concerns
Ma 2015 China [38]	RCT	Some concerns	Some concerns	Low	Low	Low	Some concerns
Wu 2023 China [41]	RCT	Some concerns	Some concerns	Low	Low	Low	Some concerns
Xia 2023 China [42]	RCT	Low	Some concerns	Low	Low	Low	Some concerns
Yang 2018 China [33]	RCT	Some concerns	High	High	Low	Low	High
Yang 2023 China [34]	RCT	Some concerns	Some concerns	Low	Low	Low	Some concerns
**(** **b)**							
**Author/Year/** **Country**	**Study Design**	**Selection (0–4)**			**Comparability (0–2)**	**Outcome (0–3)**	**Overall Risk of Bias**
Gu 2018 China [28]	quasi-experimental	3			2	3	Good
Li 2023 U.S. [31]	cohort (prospective)	3			1	3	Good
Liu 2022 China [30]	quasi-experimental	2			2	2	Fair
Rao 2021 China [39]	quasi-experimental	3			2	3	Good
Wu 2010 China [40]	quasi-experimental	2			2	2	Fair
Yan 2013 China [40]	cohort (prospective)	2			0	3	Fair
Yang 2016 China [32]	quasi-experimental	3			2	2	Good
Yang 2023 China [43]	quasi-experimental	3			2	3	Good
Zhang 2014 China [16]	cohort (prospective)	3			0	2	Fair
Zhang 2016 China [46]	cohort (prospective)	3			0	2	Fair
Zhang 2020 China [44]	quasi-experimental	3			1	2	Fair

Note. RCT = randomized controlled trial.

**Table 3:** Significant metabolites and pathways (*N* = 22).

Author/Year/ Country	Identified Metabolomic Pathways		Differential Metabolites		Statistical Analysis
	Pre vs. Post Acupuncture	Post-Acupuncture vs. Post-Sham/Alternative Intervention	Pre- vs. Post-Acupuncture	Post-Acupuncture vs. Post-Sham/Alternative Intervention	
Gao 2023 China [27]	- lactate metabolism - carbohydrate metabolism - lipid metabolism	- tRNA charging pathway - carbohydrate metabolism	- increased lactic acid - decreased acetoacetate, citric acid, pyruvic acid	- decreased glutamine	OPLS-DA, PCA
Gu 2018 China [28]	NA	NA	- increased N-acetylaspartate/creatine	NA	ANOVA, *t*-test, linear regression
Jedel 2011 Sweden [35]	NA	NA	(week 16) - decreased total testosterone, free testosterone, 5alpha-dihydrotestosterone, estrone, estrone sulfate, estradiol, dehydroepiandrosterone sulfate, 17beta-diol, androsterone glucuronide, 17beta-diol-3glucuronide, 17beta-diol-17-glucuronide (week 32) - decreased total testosterone, free testosterone, 5alpha-dihydrotestosterone, dehydroepiandrosterone sulfate, androsterone glucuronide, 17beta-diol-3glucuronide, 17beta-diol-17-glucuronide	(week 16) - decreased total testosterone, androsterone glucuronide, 17beta-diol-3glucuronide	Kruskal-Wallis test, Mann-Whitney U-test, chi-square test, Wilcoxon rank sum test
Ju 2016 China [36]	- phospholipid metabolism - tyrosine metabolism - catecholamine metabolism	NA	- increased lysophosphatidylethanolamine, estrone, uric acid, vanillylmandelic acid, N-acetyl-l-tyrosine, 4-hydroxyphenylacetylglutamine	NA	OPLS-DA
Kim 2021 South Korea [37]	NA	NA	- increased L-carnitine, butyrylcarnitine, hexanoylcarnitine, serine - decreased decanoylcarnitine, histidine	- increased acetylcarnitine, butyrylcarnitine, hexanoylcarnitine, L-carnitine	ANCOVA, *t*-test
Li 2020 China [19]	- tryptophan metabolism - glutamate metabolism - fatty acid biosynthesis	NA	- increased glutathione, N-acetyl-5-hydroxytryptamine, proline, tryptophan, cysteinylglycine - decreased malonic acid	NA	OPLS-DA, PCA
Li 2023 China [29]	- steroid hormone biosynthesis - amino acid metabolism - glycolysis - glycerophospholiqid metabolism - tryptophan metabolism	- actin cytoskeleton regulation pathway	- increased (2e,4e,12z)-n-isobutyl-2,4,12-octadecatrienamide, sphingosine, 6-methylquinoline, oglufanide, penciclovir, traumatic acid, fenipentol, 9,10-dihydroxystearic acid, ricinoleic acid, capsi-amide, (2e,4z)-n-isobutyl-2,4-octadecadienamide, 1-(14-methylhexadecanoyl)pyrrolidine, 2-decylfuran, 2-Phenylethyl octanoate, Hexadecanedioic acid, 1,3-dihydroxy-2-propanyl (9z)-9-tetradecenoate, Dibutyl decanedioate - decreased (s)-1-methoxy-3-heptanethiol, 2-oxobutyric acid, cinnamic acid, PS(15:0/22:0), 3,4-diaminopyridine, acutumidine, choline, L-proline	NA	PCA, *t*-test
Li 2023 U.S. [31]	- glutathione metabolism - arginine and proline metabolisms	NA	- increased F-1,6/2,6-DP, glutathione disulfide, phosphorylcholine, glutathione, putrescine - decreased 6-methylnicotinamide	NA	OPLS-DA, pathway analysis
Liu 2022 China [30]	- arginine metabolism - glycolysis/gluconeogenesis - riboflavin metabolism - glutamate metabolism - proline metabolism	NA	- increased alpha-D-glucose, citrulline - decreased biliverdin reductase B, flavin adenine dinucleotide, L-glutamate, enolase 1	NA	OPLS-DA
Ma 2015 China [38]	- choline metabolism - ROS stress - glycolysis - TCA cycle	NA	- increased 2-hydroxybutyrate, 3-hydroxyisovalerate, lactic acid, pyruvate, citric acid, dimethylglycine, choline, glycine, hippurate, hypoxanthine	NA	PCA, PLS-DA, OPLSDA, ANOVA
Rao 2021 China [39]	aminoacyl-tRNA biosynthesis	NA	- increased alanine, serine, homoserine, cytidine	NA	PCA, PLS-DA
Wu 2010 China [40]	NA	NA	- increased leucine/isoleucine, lactic acid - decreased glucose	NA	PCA, PLS-DA
Wu 2023 China [41]	- fatty acid metabolism - amino acid metabolism	NA	- increased glyceric acid and L-ornithine (low current intensity)	NA	OPLS-DA, *t*-test
Xia 2023 China [42]	NA	- retinol metabolism - glycine/serine/threonine metabolism - glycerophospholipid metabolism	- increased retinyl palmitate, Sn-Glycero-3-phosphocholine, LysoSM (18:0), L-cysteine, Linoleic acid, Glycocholic acid, Docosahexaenoic acid, Dihomolinoleic acid, 25-hydroxyvitamin D3 - decreased 3-Sulfopyruvic acid, 4-Oxo-retinoic acid and 3-Carboxy-4-methyl-5-propyl, Methionyl-proline, Metanephrine, Glycine, Pyruvic acid, Uridine, Phytosphingosine, Progesterone, Retinol, LysoPC (16:1), Lipoamide, Indoleacetic acid, Indole, Hydroxy Retinoic acid, Guanidinoacetic acid, Decanoylcarnitine, Acetylcholine, 3-Sulfopyruvic acid, 4-Oxo-retinoic acid and 3-Carboxy-4-methyl-5-propyl	NA	PCA, PLS-DA
Yan 2013 China [45]	- alpha-linolenic acid metabolism - D-glutamine and D-glutamate metabolism - citrate cycle - alanine metabolism - aspartate metabolism - glutamate metabolism - vitamin B6 metabolism	NA	- increased Carnitine, 3-Methylglutarylcarnitine, Hydroxyhexanoycarnitine, Sebacic acid, Supinine, Jasmonic acid, 3-Vinylcatechol, 2-Octenoylcarnitine, L-Octanoylcarnitine, L-Hexanoylcarnitine, Citric acid, Mesaconic acid, 3-Hydroxy-3-methyl-glutaric acid, 4-Pyridoxic acid, 5,7-Nonadienoic acid, Decenedioic acid, 3-tert-Butyl-5-methylcatechol - decreased alpha-N-Phenylacetyl-L-glutamine, 6Z-Octene-2,4-diynoic acid, 1-Formyl-2-indanone, 6-Keto-decanoylcarnitine, 2-trans,4-cis-Decadienoylcarnitine, 9-Decenoylcarnitine, L-Decanoylcarnitine, Androsterone glucuronide, alpha-Linolenic acid, 2-Hydroxypropyl-CoM, Dopamine 3-O-sulfate, Nicotinamide riboside, Porphobilinogen, Indoxyl sulfuric acid, 4-Sulfobenzyl alcohol, riboflavin, Suberic acid, Dodecanedioic acid, 1,8-Diazacyclotetradecane-2,9-dione, L-Glutamine, 10-oxo-decanoic acid	NA	OPLS-DA, PCA
Yang 2016 China [32]	NA	NA	- decreased oleic acid, myo-inositol	NA	PLS-DA
Yang 2018 China [33]	NA	NA	- increased hypoxanthine, hexanoic acid - decreased sucrose, cellobiose, ketoglutaric acid	- increased ketoglutaric acid	PCA, PLS-DA
Yang 2023 China [34]	- glycolytic and gluconeogenesis metabolism - TCA cycle	NA	- increased alpha-Ketoisovaleric acid, oxoadipic acid - decreased 2-Hydroxyglutaric acid, 4-Hydroxyphenylpyruvic acid, adenosine diphosphate, guanosine diphosphate, hippuric acid	- increased alpha-ketoisovaleric acid, homogentisic acid, oxoadipic acid, picolinic acid, quinolinic acid - decreased 2-hydroxyglutaric acid, 4-hydroxyphenylpyruvic acid, adenosine diphosphate, cis-aconitic acid, citric acid, flavin-adenine dinucleotide, guanosine diphosphate, hippuric acid, indoleacetic acid, isocitric acid	PCA, PLS-DA
Yang 2023 China [43]	- tryptophan metabolism - pyrimidine metabolism - lysine biosynthesis	NA	- increased 4-hydroxy-5-(dihydroxyphenyl)-valeric acid-O-methyl-O-sulphate, 3-(3-hydroxyphenyl)-2-phenyl-4-[(E)-2-phenylethenyl]-2,3-dihydro-1-benzofuran-6-ol, proacaciberin, 1-hydroxy-3-methoxy-10-methylacridone - decreased desethyletomidate, OOV-PE	NA	OPLS-DA
Zhang 2014 China [16]	- phenylalanine metabolism - alanine metabolism - aspartate metabolism - glutamate metabolism - D-glutamine and D-glutamate - steroid hormone biosynthesis	NA	- increased phenylpyruvic acid, Buthidazole, 7-Hydroxyondansetron glucuronide, 6-Hydroxyondansetron glucuronide, tryptophyltryptophan, xestoaminol C, 17beta-Hydroxy-4,17-dimethyl-4-azaandrost-5-en-3-one, N-Oleoyl threonine, Tiamulin, N,2,3-Trimethyl-2-(1-methylethyl)butanamide, clavamycin A, L-Phenylalanyl-L-tyrosine, (S)-Spirobrassinin, 5-Sulfosalicylic acid, dorspoinsettifolin, norharman, Semilepidinoside A - decreased anastrozole, LPE 0:0/20:3, butoctamide hydrogen succinate, C17 Sphinganine, 17,21-Dihydroxypregn-4-ene-3,11,20-trione 21-(hydrogensuccinate), 6-Hydroxy-9Z,12Z-octadecadienoic acid, 8-Hydroxy-11Z-octadecen-9-ynoic acid, 5-hydroxyeicosatetraenoic acid	NA	PCA, PLS-DA
Zhang 2016 China [46]	- glycerophospholipid metabolism - ether lipid metabolism - fatty acid metabolism - glycerolipid metabolism - porphyrin metabolism - sphingolipid metabolism - primary bile acid biosynthesis - fatty acid elongation in mitochondria - fatty acid biosynthesis - tryptophan metabolism	- phenylalanine metabolism - alanine metabolism - aspartate metabolism - glutamate metabolism - D-glutamine and D-glutamate metabolism - steroid hormone biosynthesis pathways	- increased glycerophosphocholine, sn-glycero-3-Phosphocholine, LPC 18:2, LPC P-16:0, LPC P-18:1, LPCP-18:0, LPC O-18:0, PC 34:2, PC 36:2, SM d18:0, PC 34:2, PC 36:2, LPE P-16:0, LPC P-16:0, LPE 17:2, LPC 0:0 - decreased sphinganine, 2-amino-tridecanoic acid, anandamide (20:2, n-6), 2-amino-14,16-dimethyloctadecan-3-ol, MG 16:0, 3-oxohexacosanoic acid, MG 24:1, MG 18:0, indoxyl sulfate, LPC 14:0, LPE 22:6, LPE 18:2, LPE 20:4, LPC 0:0, LPC 16:0, LPE 18:1, LPC 17:0, LPC 18:0	NA	PCA, PLS-DA
Zhang 2020 China [44]	- propanoate metabolism - butanoate metabolism - TCA cycle	NA	- increased psicose, D-mannitol - decreased butantriol, 3,4-dihydroxybutanoic acid, succinic acid, 1-deoxypentitol, citric acid, 3-hydroxybutyric acid and hydracrylic acid,3-hydroxyphenylacetic acid	NA	OPLS-DA

Note. ANCOVA = analysis of covariance; ANOVA = analysis of variance; NA = not assessed; (O)PLS-DA = (orthogonal signal correction for) partial least square discriminate analysis; PCA = principal component analysis; TCA = tricarboxylic acid; tRNA = transfer ribonucleic acid.

## Data Availability

The data that support the findings of this study are openly available in the manuscript and Supplementary Materials. Details of the included studies in this review are reported in the References and the PROSPERO registration (CRD42024503656).

## Supplementary Materials

The following supporting information can be downloaded at: https://www.mdpi.com/article/10.3390/metabo14100542/s1, Supplementary Table S1: search strategies; Supplementary Table S2: significant metabolites across studies; Supplementary Table S3: pathway analysis result for 226 significant metabolites; Supplementary Table S4: pathway analysis result for 14 common significant metabolites; Table S5: PRISMA 2020 checklist: reporting guidelines for systematic review. Reference [21] is cited in the supplementary material Table S5.
