# Supplementary material for: Impact of Acupuncture on Human Metabolomic Profiles: A Systematic Review
Source: Metabolites. 2024 Oct 11;14(10):542. doi: 10.3390/metabo14100542 (PMC11509109; doi:10.3390/metabo14100542)
Supplement: Supplementary file 1 [file metabolites-14-00542-s001.zip › metabolites-3210420-supplementary.pdf]

Supplementary Materials

Table S1. Search strategies

Database	Search strategy
PubMed	("Acupuncture Therapy"[MeSH Terms:noexp] OR "Acupuncture Analgesia"[MeSH Terms] OR "acupuncture, ear"[MeSH Terms] OR "Electroacupuncture"[MeSH Terms] OR "Acupuncture"[MeSH Terms] OR "acupunctur*"[Title/Abstract] OR "pharmacoacupunctur*"[Title/Abstract] OR "pharmaco acupunctur*"[Title/Abstract] OR "acupotom*"[Title/Abstract] OR "auriculoacupunctur*"[Title/Abstract] OR "auriculotherap*"[Title/Abstract] OR "Needle Moxibustion"[Title/Abstract] OR "acupoint*"[Title/Abstract] OR "needling"[Title/Abstract] OR "trigger point*"[Title/Abstract])<br>AND (metabolite*[Title/Abstract] OR metabolomic*[Title/Abstract] OR "metabolite marker*" [Title/Abstract] OR "metabolic profile*"[Title/Abstract] OR metabolism*[Title/Abstract] OR biomarker*[Title/Abstract] OR "biological marker*" [Title/Abstract] OR endophenotype*[Title/Abstract] OR "Metabolome"[MeSH Terms] OR "Metabolomics"[MeSH Terms] OR "Biomarkers"[MeSH Terms])
Embase	('acupuncture'/exp OR 'acupuncture analgesia'/exp OR 'auricular acupuncture'/exp OR 'electroacupuncture'/exp OR "acupunctur*":ti,ab OR 'pharmacopuncture':ti,ab OR "acupotom*":ti,ab OR "auriculoacupunctur*":ti,ab OR "auriculotherap*":ti,ab OR "Needle Moxibustion":ti,ab OR "acupoint*":ti,ab OR "needling":ti,ab OR "trigger point*":ti,ab)<br>AND ('metabolomics'/exp OR 'biological marker'/exp OR "metabolite*":ti,ab OR "metabolomic*":ti,ab OR "metabolite marker":ti,ab OR "metabolic profile":ti,ab OR "metabolism*":ti,ab OR "biomarker*":ti,ab OR "biological marker":ti,ab OR "endophenotype*":ti,ab)
Scopus	(TITLE-ABS ("Acupuncture Therapy") OR TITLE-ABS ("Acupuncture, Ear") OR TITLE-ABS ("Acupuncture") OR TITLE-ABS ("Dry Needling") OR TITLE-ABS ("Acupuncture Points") OR TITLE-ABS ("Acupuncture Analgesia") OR TITLE-ABS ("Electroacupuncture") OR TITLE-ABS (acupunctur*) OR TITLE-ABS ("Acupuncture Therapy") OR TITLE-ABS (electroacupunctur*) OR TITLE-ABS (pharmacoacupunctur*) OR TITLE-ABS (acupotom*) OR TITLE-ABS (auriculoacupunctur*) OR TITLE-ABS (auriculotherap*) OR TITLE-ABS ("Needle Moxibustion") OR TITLE-ABS (acupoint*) OR TITLE-ABS (needling) OR TITLE-ABS ("trigger point"))<br>AND (TITLE-ABS ("Metabolomics") OR TITLE-ABS ("Biomarkers") OR TITLE-ABS (metabolite*) OR TITLE-ABS (metabolomic*) OR TITLE-ABS ("metabolite marker") OR TITLE-ABS ("metabolic profile") OR TITLE-ABS (metabolism*) OR TITLE-ABS (biomarker*) OR TITLE-ABS ("biological marker") OR TITLE-ABS (endophenotype*))

CINAHL	((MH "Acupuncture+") OR (MH "Dry Needling") OR (MH "Acupuncture, Ear") OR (MH "Acupuncture Points") OR (MH "Acupuncture Anesthesia") OR (MH "Acupuncture Analgesia") OR (MH "Electroacupuncture") OR TI"Acupuncture Therapy" OR AB"Acupuncture Therapy" OR TI"Electroacupuncture" OR AB"Electroacupuncture" OR TI pharmacoacupunctur* OR AB pharmacoacupunctur* OR TI acupunctur* OR AB acupunctur* OR TI "pharmaco acupunctur*" OR AB "pharmaco acupunctur*" OR TI "acupotom*" OR AB "acupotom*" OR TI auriculoacupunctur* OR AB auriculoacupunctur* OR TI auriculotherap* OR AB auriculotherap* OR TI "Needle Moxibustion" OR AB "Needle Moxibustion" OR TI acupoint* OR AB acupoint* OR TI needling OR AB needling OR TI "trigger point*" OR AB "trigger point*")<br>AND (TI metabolite* OR AB metabolite* OR TI metabolomic* OR AB metabolomic* OR TI 'metabolite marker*' OR AB 'metabolite marker*' OR TI 'metabolic profile*' OR AB 'metabolic profile*' OR TI metabolism* OR AB metabolism* OR TI biomarker* OR AB biomarker* OR TI 'biological marker*' OR AB 'biological marker*' OR TI endophenotype* OR AB endophenotype* OR (MH "Metabolomics") OR (MH "Biological Markers+"))
Cochrane Central Register of Controlled Trials	(MeSH descriptor: [Acupuncture Therapy] explode all trees OR MeSH descriptor: [Acupuncture, Ear] explode all trees OR MeSH descriptor: [Acupuncture] explode all trees OR MeSH descriptor: [Dry Needling] explode all trees OR MeSH descriptor: [Acupuncture Points] explode all trees OR MeSH descriptor: [Acupuncture Analgesia] explode all trees OR MeSH descriptor: [Electroacupuncture] explode all trees OR (acupunctur*):ti,ab,kw OR ("Acupuncture Therapy"):ti,ab,kw OR (Electroacupunctur*):ti,ab,kw OR (pharmacoacupunctur*):ti,ab,kw OR (acupotom*):ti,ab,kw OR (auriculoacupunctur*):ti,ab,kw OR (auriculotherap*):ti,ab,kw OR ("Needle Moxibustion"):ti,ab,kw OR (acupoint*):ti,ab,kw OR (needling):ti,ab,kw OR ("trigger point"):ti,ab,kw)<br>AND (MeSH descriptor: [Metabolomics] in all MeSH products OR MeSH descriptor: [Biomarkers] explode all trees OR (metabolite*):ti,ab,kw OR (metabolomic*):ti,ab,kw OR ("metabolite marker"):ti,ab,kw OR (“metabolic profile”):ti,ab,kw OR (metabolism*):ti,ab,kw OR (biomarker*):ti,ab,kw OR (“biological marker”):ti,ab,kw OR (endophenotype*):ti,ab,kw)

Table S2. Significant metabolites across studies (n=226)

Metabolites	Study
**(2e,4e,12z)-n-isobutyl-2,4,12-octadecatrienamide**	Li 2023a
**(2e,4z)-n-isobutyl-2,4-octadecadienamide**	Li 2023a
**(S)-1-methoxy-3-heptanethiol**	Li 2023a

**(S)-Spirobrassinin**	Zhang 2014
**{[6-(5,7-dihydroxy-4-oxo-2-phenyl-3,4-dihydro-2H-1-benzopyran-8-yl)-3,4,5-trihydroxyoxan-2-yl]methoxy}sulfonic acid**	Yang 2023b
**1-(14-methylhexadecanoyl)pyrrolidine**	Li 2023a
**1,3-Dihydroxy-2-propanyl (9z)-9-tetradecenoate**	Li 2023a
**1,8-Diazacyclotetradecane-2,9-dione**	Yan 2013
**10-oxo-decanoic acid**	Yan 2013
**17,21-Dihydroxypregn-4-ene-3,11,20-trione 21-(hydrogensuccinate)**	Zhang 2014
**17beta-diol-3glucuronide**	Jedel 2011
**17beta-Hydroxy-4,17-dimethyl-4-azaandrost-5-en-3-one**	Zhang 2014
**1-Deoxypentitol**	Zhang 2020
**1-Formyl-2-indanone**	Yan 2013
**1-Hydroxy-3-methoxy-10-methylacridone**	Yang 2023b
**25-Hydroxyvitamin D3**	Xia 2023
**2-Amino-14,16-dimethyloctadecan-3-ol**	Zhang 2016
**2-Amino-tridecanoic acid**	Zhang 2016
**2-Decylfuran**	Li 2023a
**2-Hydroxybutyrate**	Ma 2015
**2-Hydroxyglutaric acid**	Yang 2023a
**2-Hydroxypropyl-CoM**	Yan 2013
**2-Octenoylcarnitine**	Yan 2013
**2-Oxobutyric acid**	Li 2023a
**2-Phenylethyl octanoate**	Li 2023a
**2-Trans,4-cis-Decadienoylcarnitine**	Yan 2013
**3-(3-hydroxyphenyl)-2-phenyl-4-[(E)-2-phenylethenyl]-2,3-dihydro-1-benzofuran-6-ol**	Yang 2023b
**3,4-Diaminopyridine**	Li 2023a
**3,4-Dihydroxybutanoic acid**	Zhang 2020
**3-Carboxy-4-methyl-5-propyl**	Xia 2023
**3-Hydroxy-3-methyl-glutaric acid**	Yan 2013
**3-Hydroxybutyric acid**	Zhang 2020
**3-Hydroxyisovalerate**	Ma 2015
**3-Hydroxyphenylacetic acid**	Zhang 2020

**3-Methylglutarylcarnitine**	Yan 2013
**3-Oxohexacosanoic acid**	Zhang 2016
**3-Sulfopyruvic acid**	Xia 2023
**3-Tert-Butyl-5-methylcatechol**	Yan 2013
**3-Vinylcatechol**	Yan 2013
**4-Hydroxy-5-(dihydroxyphenyl)-valeric acid-O-methyl-O-sulphate**	Yang 2023b
**4-Hydroxyphenylacetylglutamine**	Ju 2016
**4-Hydroxyphenylpyruvic acid**	Yang 2023a
**4-Oxo-retinoic acid**	Xia 2023
**4-Pyridoxic acid**	Yan 2013
**4-Sulfobenzyl alcohol**	Yan 2013
**5,7-Nonadienoic acid**	Yan 2013
**5-Hydroxyeicosatetraenoic acid**	Zhang 2014
**5-Sulfosalicylic acid**	Zhang 2014
**6-Hydroxy-9Z,12Z-octadecadienoic acid**	Zhang 2014
**6-Hydroxyondansetron glucuronide**	Zhang 2014
**6-Keto-decanoylcarnitine**	Yan 2013
**6-Methylnicotinamide**	Li 2023b
**6-Methylquinoline**	Li 2023a
**6Z-Octene-2,4-diynoic acid**	Yan 2013
**7-Hydroxyondansetron glucuronide**	Zhang 2014
**8-Hydroxy-11Z-octadecen-9-ynoic acid**	Zhang 2014
**9,10-Dihydroxystearic acid**	Li 2023a
**9-Decenoylcarnitine**	Yan 2013
**Acetoacetate**	Gao 2023
**Acetylcholine**	Xia 2023
**Acutumidine**	Li 2023a
**Adenosine diphosphate**	Yang 2023a
**Alanine**	Rao 2021
**Alpha-D-glucose**	Liu 2022
**Alpha-Ketoisovaleric acid**	Yang 2023a

**Alpha-Linolenic acid**	Yan 2013
**Alpha-N-Phenylacetyl-L-glutamine**	Yan 2013
**Anandamide (20:2, n-6)**	Zhang 2016
**Anastrozole**	Zhang 2014
**Androsterone glucuronide**	Jedel 2011, Yan 2013
**Biliverdin reductase B (BLVRB)**	Liu 2022
**Butantriol**	Zhang 2020
**Buthidazole**	Zhang 2014
**Butoctamide hydrogen succinate**	Zhang 2014
**Butyrylcarnitine**	Kim 2021
**C17 Sphinganine**	Zhang 2014
**Capsi-amide**	Li 2023a
**Carnitine**	Yan 2013
**Cellobiose**	Yang 2018
**Choline**	Li 2023a, Ma 2015
**Cinnamic acid**	Li 2023a
**Citrate**	Gao 2023, Ma 2015
**Citric acid**	Yan 2013, Zhang 2020
**Citrulline**	Liu 2022, Yang 2016
**Clavamycin A**	Zhang 2014
**Cysteine**	Xia 2023
**Cysteinylglycine**	Li 2020
**Cytidine**	Rao 2021
**D-(+)-galactose**	Yang 2016
**Decanoylcarnitine**	Kim 2021, Xia 2013
**Decenedioic acid**	Yan 2013
**Desethyletomidate**	Yang 2023b
**Dibutyl decanedioate**	Li 2023a
**Dihomolinoleic acid**	Xia 2023
**Dimethylglycine**	Ma 2015
**D-mannitol**	Zhang 2020

**Docosahexaenoic acid**	Xia 2023
**Dodecanedioic acid**	Yan 2013
**Dopamine 3-O-sulfate**	Yan 2013
**Dorspoinsettifolin**	Zhang 2014
**Enolase 1 (ENO1)**	Liu 2022
**Estrone**	Ju 2016
**F-1,6/2,6-DP**	Li 2023b
**Fenipentol**	Li 2023a
**Flavin adenine dinucleotide (FAD)**	Liu 2022
**Fructose**	Yang 2016
**Glucose**	Wu 2010
**Glutamate**	Liu 2022
**Glutamine**	Yang 2013, Gao 2023
**Glutathione**	Li 2020, Li 2023b
**Glutathione disulfide**	Li 2023b
**Glyceric acid**	Wu 2023
**Glycerophosphocholine**	Zhang 2016
**Glycine**	Ma 2015, Yang 2016, Xia 2013
**Glycocholic acid**	Xia 2023
**Guanidinoacetic acid**	Xia 2023
**Guanosine diphosphate**	Yang 2023a
**Hexadecanedioic acid**	Li 2023a
**Hexanoic acid**	Yang 2018
**Hexanoylcarnitine**	Kim 2021
**Hippurate**	Ma 2015
**Hippuric acid**	Yang 2023a
**Histidine**	Kim 2021
**Homoserine**	Rao 2021
**Hydracrylic acid**	Zhang 2020
**Hydroxy Retinoic acid**	Xia 2023
**Hydroxyhexanoycarnitine**	Yan 2013

**Hypoxanthine**	Ma 2015, Yang 2018
**Indole**	Xia 2023
**Indoleacetic acid**	Xia 2023
**Indoxyl sulfate**	Zhang 2016
**Indoxyl sulfuric acid**	Yan 2013
**Jasmonic acid**	Yan 2013
**Ketoglutaric acid**	Yang 2018
**Lactate**	Ma 2015, Wu 2010
**Lactic acid**	Gao 2023
**L-carnitine**	Kim 2021
**L-Decanoylcarnitine**	Yan 2013
**Leucine/isoleucine**	Wu 2010
**L-hexanoylcarnitine**	Yan 2013
**Linoleic acid**	Xia 2023
**Lipoamide**	Xia 2023
**L-octanoylcarnitine**	Yan 2013
**L-ornithine**	Wu 2023
**Lysopc (16:1)**	Xia 2023
**Lysopc(0:0/16:0)**	Zhang 2016
**Lysopc(0:0/P-18:0)**	Zhang 2016
**Lysopc(14:0)**	Zhang 2016
**Lysopc(16:0/0:0)**	Zhang 2016
**Lysopc(17:0)**	Zhang 2016
**Lysopc(18:0)**	Zhang 2016
**Lysopc(18:2(9Z,12Z))**	Zhang 2016
**Lysopc(O-18:0)**	Zhang 2016
**LysoPC(P-16:0)**	Zhang 2016
**LysoPC(P-16:0)**	Zhang 2016
**LysoPC(P-18:0/0:0)**	Zhang 2016
**LysoPC(P-18:1(9Z))**	Zhang 2016
**LysoPE(0 : 0/20 : 3(8Z,11Z,14Z))**	Zhang 2014

**LysoPE(17:2)**	Zhang 2016
**LysoPE(18:1(11Z)/0:0)**	Zhang 2016
**LysoPE(18:2(9Z,12Z)/0:0)**	Zhang 2016
**LysoPE(20:4(8Z,11Z,14Z,17Z)/0:0)**	Zhang 2016
**LysoPE(22:6(4Z,7Z,10Z,13Z,16Z,19Z)/0:0)**	Zhang 2016
**LysoPE(P-16:0/0:0)**	Zhang 2016
**Lysophosphatidylethanolamine**	Ju 2016
**Lysosm (18:0)**	Xia 2023
**Malonic acid**	Li 2020
**Mesaconic acid**	Yan 2013
**Metanephrine**	Xia 2023
**Methionyl-proline**	Xia 2023
**MG(16:0/0:0/0:0)**	Zhang 2016
**MG(18:0/0:0/0:0)**	Zhang 2016
**MG(24:1(15Z)/0:0/0:0)**	Zhang 2016
**Myo-inositol**	Yang 2016
**N,2,3-Trimethyl-2-(1-methylethyl)butanamide**	Zhang 2014
**N-Acetyl-5-hydroxytryptamine**	Li 2020
**N-Acetylaspartate/creatine**	Gu 2018
**N-Acetyl-l-tyrosine**	Ju 2016
**Nicotinamide riboside**	Yan 2013
**N-Oleoyl threonine**	Zhang 2014
**Norharman**	Zhang 2014
**Oglufanide**	Li 2023a
**Oleic acid**	Yang 2016
**1,2-Diacyl-sn-glycero-3-phosphoethanolamine (OOV-PE)**	Yang 2023b
**Oxoadipic acid**	Yang 2023a
**PC 34:2**	Zhang 2016
**PC 34:2**	Zhang 2016
**PC 36:2**	Zhang 2016
**PC 36:2**	Zhang 2016

**Penciclovir**	Li 2023a
**Phenylpyruvic acid**	Zhang 2014
**L-Phenylalanyl-L-tyrosine (Phe-Tyr-OH)**	Zhang 2014
**Phosphorylcholine**	Li 2023b
**Phytosphingosine**	Xia 2023
**Porphobilinogen**	Yan 2013
**proacaciberin**	Yang 2023b
**Progesterone**	Xia 2023
**Proline**	Li 2020; Li 2023a
**Ps(15:0/22:0)**	Li 2023a
**Psicose**	Zhang 2020
**Putrescine**	Li 2023b
**Pyruvate**	Ma 2015
**Pyruvic acid**	Gao 2023; Xia 2023
**Retinol**	Xia 2023
**Retinyl palmitate**	Xia 2023
**Riboflavin**	Yan 2013
**Ricinoleic acid**	Li 2023a
**Sebacic acid**	Yan 2013
**Semilepidinoside A**	Zhang 2014
**Serine**	Kim 2021, Rao 2021
**SM(d18:0/16:1(9Z))**	Zhang 2016
**Sn-glycero-3-Phosphocholine**	Zhang 2016, Xia 2023
**Sphinganine**	Zhang 2016
**Sphingosine**	Li 2023a
**Succinic acid**	Zhang 2020
**Sucrose**	Yang 2018
**Supinine**	Yan 2013
**Testosterone**	Jedel 2011
**Tryptophyltryptophan**	Zhang 2014
**Tiamulin**	Zhang 2014

**Traumatic acid**	Li 2023a
**Tryptophan**	Li 2020
**Uric acid**	Ju 2016
**Uridine**	Xia 2023
**Vanillylmandelic acid**	Ju 2016
**Xestoaminol C**	Zhang 2014

Table S3. Pathway analysis result for 226 significant metabolites

Pathway Name	Match Status	p	-log(p)	Holm p	FDR	Impact
**Glycine, serine and threonine metabolism**	9/33	9.4656E-6	5.0239	7.5725E-4	7.5725E-4	0.60014
**Glutathione metabolism**	7/28	1.865E-4	3.7293	0.014733	0.0074599	0.43909
**Glyoxylate and dicarboxylate metabolism**	6/32	0.0027964	2.5534	0.21812	0.059759	0.25927
**Arginine biosynthesis**	4/14	0.0029879	2.5246	0.23007	0.059759	0.28934
**Butanoate metabolism**	4/15	0.0039328	2.4053	0.29889	0.062925	0.11111
**Galactose metabolism**	5/27	0.0067651	2.1697	0.50739	0.079428	0.10311
**Starch and sucrose metabolism**	4/18	0.0079305	2.1007	0.58686	0.079428	0.4755
**Alanine, aspartate and glutamate metabolism**	5/28	0.0079428	2.1	0.58686	0.079428	0.16186
**Citrate cycle (TCA cycle)**	4/20	0.011703	1.9317	0.84264	0.095746	0.22801
**Phenylalanine, tyrosine and tryptophan biosynthesis**	2/4	0.011968	1.922	0.84974	0.095746	0.0
**Cysteine and methionine metabolism**	5/33	0.01601	1.7956	1.0	0.10981	0.17854
**Propanoate metabolism**	4/22	0.016472	1.7833	1.0	0.10981	0.04103
**Arginine and proline metabolism**	5/36	0.022826	1.6416	1.0	0.14047	0.41511
**Lipoic acid metabolism**	4/28	0.037392	1.4272	1.0	0.21367	0.0017
**Tyrosine metabolism**	5/42	0.041535	1.3816	1.0	0.22152	0.08487
**Valine, leucine and isoleucine biosynthesis**	2/8	0.049501	1.3054	1.0	0.2475	0.0
**Sphingolipid metabolism**	4/32	0.057169	1.2428	1.0	0.26903	0.14154

Table S4. Pathway analysis result for 15 common significant metabolites

Pathway Name	Match Status	p	-log(p)	Holm p	FDR	Impact
**Glyoxylate and dicarboxylate metabolism**	5/32	2.8771E-6	5.541	2.3017E-4	2.3017E-4	0.1799
**Glycine, serine and threonine metabolism**	4/33	1.002E-4	3.9991	0.0079156	0.0040079	0.4744
**Alanine, aspartate and glutamate metabolism**	3/28	0.0012788	2.8932	0.099748	0.034102	0.11378
**Arginine biosynthesis**	2/14	0.0054143	2.2665	0.4169	0.10829	0.22843
**Citrate cycle (TCA cycle)**	2/20	0.010993	1.9589	0.83544	0.17588	0.13672
**Pyruvate metabolism**	2/23	0.014434	1.8406	1.0	0.18728	0.19137
**Glycolysis/ Gluconeogenesis**	2/26	0.018285	1.7379	1.0	0.18728	0.09785
**Lipoic acid metabolism**	2/28	0.021069	1.6764	1.0	0.18728	0.0017
**Glutathione metabolism**	2/28	0.021069	1.6764	1.0	0.18728	0.34469
**Cysteine and methionine metabolism**	2/33	0.028753	1.5413	1.0	0.22554	0.02184
**Arginine and proline metabolism**	2/36	0.033832	1.4707	1.0	0.22554	0.01744
**Glycerophospholipid metabolism**	2/36	0.033832	1.4707	1.0	0.22554	0.07396

Table S5. PRISMA 2020 Checklist: Reporting guidelines for Systematic review

Section and Topic	Item #	Checklist item	Location where item is reported
**TITLE**			
Title	1	Identify the report as a systematic review.	p.1
**ABSTRACT**			
Abstract	2	See the PRISMA 2020 for Abstracts checklist.	p.1
**INTRODUCTION**			
Rationale	3	Describe the rationale for the review in the context of existing knowledge.	p.1-2
Objectives	4	Provide an explicit statement of the objective(s) or question(s) the review addresses.	p.2
**METHODS**			
Eligibility criteria	5	Specify the inclusion and exclusion criteria for the review and how studies were grouped for the syntheses.	p.2-3
Information sources	6	Specify all databases, registers, websites, organisations, reference lists and other sources searched or consulted to identify studies. Specify the date when each source was last searched or consulted.	p.3-4
Search strategy	7	Present the full search strategies for all databases, registers and websites, including any filters and limits used.	p.3-4, Table S1
Selection process	8	Specify the methods used to decide whether a study met the inclusion criteria of the review, including how many reviewers screened each record and each report retrieved, whether they worked independently, and if applicable, details of automation tools used in the process.	p.3-4
Data collection process	9	Specify the methods used to collect data from reports, including how many reviewers collected data from each report, whether they worked independently, any processes for obtaining or confirming data from study investigators, and if applicable, details of automation tools used in the process.	p.3-4
Data items	10a	List and define all outcomes for which data were sought. Specify whether all results that were compatible with each outcome domain in each study were sought (e.g. for all measures, time points, analyses), and if not, the methods used to decide which results to collect.	p.3-5
	10b	List and define all other variables for which data were sought (e.g. participant and intervention characteristics, funding sources). Describe any assumptions made about any missing or unclear information.	p.3-5
Study risk of bias assessment	11	Specify the methods used to assess risk of bias in the included studies, including details of the tool(s) used, how many reviewers assessed each study and whether they worked independently, and if applicable, details of automation tools used in the process.	p.5

Section and Topic	Item #	Checklist item	Location where item is reported
Effect measures	12	Specify for each outcome the effect measure(s) (e.g. risk ratio, mean difference) used in the synthesis or presentation of results.	N/A
Synthesis methods	13a	Describe the processes used to decide which studies were eligible for each synthesis (e.g. tabulating the study intervention characteristics and comparing against the planned groups for each synthesis (item #5)).	p.4-5
	13b	Describe any methods required to prepare the data for presentation or synthesis, such as handling of missing summary statistics, or data conversions.	p.4-5
	13c	Describe any methods used to tabulate or visually display results of individual studies and syntheses.	p.4-5
	13d	Describe any methods used to synthesize results and provide a rationale for the choice(s). If meta-analysis was performed, describe the model(s), method(s) to identify the presence and extent of statistical heterogeneity, and software package(s) used.	p.4-5
	13e	Describe any methods used to explore possible causes of heterogeneity among study results (e.g. subgroup analysis, meta-regression).	N/A
	13f	Describe any sensitivity analyses conducted to assess robustness of the synthesized results.	N/A
Reporting bias assessment	14	Describe any methods used to assess risk of bias due to missing results in a synthesis (arising from reporting biases).	p.4-5
Certainty assessment	15	Describe any methods used to assess certainty (or confidence) in the body of evidence for an outcome.	N/A
**RESULTS**			
Study selection	16a	Describe the results of the search and selection process, from the number of records identified in the search to the number of studies included in the review, ideally using a flow diagram.	p.5, Figure 1
	16b	Cite studies that might appear to meet the inclusion criteria, but which were excluded, and explain why they were excluded.	p.5, Figure 1
Study characteristics	17	Cite each included study and present its characteristics.	p.5-9, Table 1
Risk of bias in studies	18	Present assessments of risk of bias for each included study.	p.10-11, Table 2
Results of individual studies	19	For all outcomes, present, for each study: (a) summary statistics for each group (where appropriate) and (b) an effect estimate and its precision (e.g. confidence/credible interval), ideally using structured tables or plots.	p.11-18, Table 3

Section and Topic	Item #	Checklist item	Location where item is reported
Results of syntheses	20a	For each synthesis, briefly summarise the characteristics and risk of bias among contributing studies.	p.11-18, Figure 2
	20b	Present results of all statistical syntheses conducted. If meta-analysis was done, present for each the summary estimate and its precision (e.g. confidence/credible interval) and measures of statistical heterogeneity. If comparing groups, describe the direction of the effect.	N/A
	20c	Present results of all investigations of possible causes of heterogeneity among study results.	p.22-23
	20d	Present results of all sensitivity analyses conducted to assess the robustness of the synthesized results.	N/A
Reporting biases	21	Present assessments of risk of bias due to missing results (arising from reporting biases) for each synthesis assessed.	p.22-23
Certainty of evidence	22	Present assessments of certainty (or confidence) in the body of evidence for each outcome assessed.	N/A
**DISCUSSION**			
Discussion	23a	Provide a general interpretation of the results in the context of other evidence.	p.20-23
	23b	Discuss any limitations of the evidence included in the review.	p.22-23
	23c	Discuss any limitations of the review processes used.	p.22-23
	23d	Discuss implications of the results for practice, policy, and future research.	p.20-23
**OTHER INFORMATION**			
Registration and protocol	24a	Provide registration information for the review, including register name and registration number, or state that the review was not registered.	p.3
	24b	Indicate where the review protocol can be accessed, or state that a protocol was not prepared.	p.3
	24c	Describe and explain any amendments to information provided at registration or in the protocol.	N/A
Support	25	Describe sources of financial or non-financial support for the review, and the role of the funders or sponsors in the review.	p.24
Competing interests	26	Declare any competing interests of review authors.	p.24
Availability of data, code and other materials	27	Report which of the following are publicly available and where they can be found: template data collection forms; data extracted from included studies; data used for all analyses; analytic code; any other materials used in the review.	p.24

Note. From: Page MJ, McKenzie JE, Bossuyt PM, Boutron I, Hoffmann TC, Mulrow CD, et al. The PRISMA 2020 statement: an updated guideline for reporting systematic reviews. BMJ 2021;372:n71. doi: 10.1136/bmj.n71
